# Simplified *Plasmodium falciparum* membrane feeding assay using small Petri dishes and gel warmers

**DOI:** 10.1186/s12936-024-05226-0

**Published:** 2024-12-22

**Authors:** Godfree Mlambo, Tassanee Thanakornsombut, Abhai K. Tripathi

**Affiliations:** 1 Johns Hopkins Malaria Research Institute, Baltimore, MD 21205 USA; 2 Department of Molecular Microbiology and Immunology, Johns Hopkins Bloomberg School of Public Health, 615 N. Wolfe Street, Baltimore, MD 21205 USA

**Keywords:** *Plasmodium falciparum*, Gametocytes, Standard membrane feeding assays, SMFA, Malaria

## Abstract

Studies on *Plasmodium falciparum* transmission require blood-feeding infectious gametocytes to mosquitoes using standard membrane-feeding assays (SMFAs). SMFAs are routinely performed using electric heating coils or glass membrane feeders connected to a circulatory water bath using tubing and clamps. Each of these approaches is expensive and requires a complex setup, hence restricting the number of assays that can be performed simultaneously. Furthermore, existing methods cannot be easily applied in low-resource field settings. This study presents a low-cost and simplified method for feeding mosquitoes with an infectious blood meal using 35 mm Petri dishes where temperature is maintained by using reusable gel warmers. The intensity and prevalence of infection in mosquitoes (*Anopheles stephensi* and *Anopheles gambiae*) fed via a Petri dish overlaid with gel warmers were comparable to mosquitoes fed using standard glass membrane feeders. The methodology described here can be easily applied in low-resource and field settings due to its low cost, ease of set up, and use of easily available supplies, such as Petri dishes, and reusable gel warmers. We believe a wide range of laboratories can easily adapt this method for *P. falciparum* transmission studies.

## Background

Malaria is a major public health problem due to the mortality and morbidity it causes, particularly in poor parts of the world. According to the 2023 WHO Malaria report, 249 million cases were recorded and 94% of these cases were in Africa [1]. Countries in Africa accounted for 95% of all deaths attributable to malaria [1]. Malaria disproportionately affects low to medium-income countries. This hampers efforts to collect data and conduct meaningful research studies aimed at reducing disease burden in these low to medium-income countries. Donor funding has been instrumental in supporting malaria research in most developing countries. However, research supported by donor funding is limited to time and priorities determined by donor-supporting agencies and is often not sustainable in the long term [2]. There is a critical need for new tools, particularly low-cost methodologies that can be sustainably used in resource-limited countries to support locally funded projects [3]. To be effective, malaria control or elimination programs must be supported by data derived from malaria-endemic regions that bear the burden of malaria. In addition to developing methodologies for use in resource-limited countries, there is a need for tools that can be easily adapted for field studies.

Malaria parasite transmission from humans to mosquitoes requires the presence of mature gametocytes in the peripheral circulation of human hosts. Membrane feeding assays (MFAs) are used to evaluate the likelihood of *Plasmodium* transmission from gametocyte-infected blood to mosquitoes. These assays can be either direct membrane feeding assays (DMFAs), which use venous blood from patients, or standard membrane feeding assays (SMFAs), which employ in vitro cultured gametocytes. SMFAs are used to assess the potential of transmission-blocking interventions such as drugs, antibodies, and genetically modified mosquitoes [4–8]. These assays are also very important in the assessment of transmission dynamics in malaria-endemic countries [9, 10]. SMFA are conducted using jacketed glass membrane feeders connected to a circulatory water bath to maintain the infectious blood feed at 37 °C to prevent premature gametogenesis [11, 12]. One alternative to using glass membrane feeders is the Hemotek feeders where the temperature of each feeder is electronically regulated by a heating element [13, 14]. Establishing a Hemotek feeding system in a laboratory is highly expensive; for example, a set-up for feeding up to 6 mosquito cups at a time costs at least US $3000 [15]. While the Hemotek feeders could be an alternative to the glass membrane feeder, they have not been widely used in transmission studies because lower infection rates have been observed when using this method [16]. Even though glass membrane feeders coupled to a circulatory water bath may be more affordable than the Hemotek system, the financial burden of setting up the glass membrane feeder can still be challenging for laboratories in low-resource countries.

During DMFA, blood samples are frequently transported from sample collection sites to an insectary that may be far away from the field. This is necessary for feeding the mosquitoes with venous blood from humans. Transporting samples from the field site to the laboratory can sometimes lead to a compromise in their quality. In their study, Soumare et al*.* [17] outlined a technique for preserving the infectivity of *P. falciparum* gametocytes using thermos flasks. Using this method, gametocyte infectivity can be preserved for up to 4 h [17]. The simplified MFA method described here enables the use of fresh blood samples directly from patients to be fed to mosquitoes without requiring transportation to the laboratory, provided it is feasible to bring mosquitoes to the field site. Methodologies supporting membrane feeding assays, whether in laboratory or field conditions, should consider that field-caught mosquitoes may need adaptation to feed through an artificial membrane, such as parafilm. Notably, variations in infection intensity have been observed between mosquitoes maintained in laboratory colonies and those captured in the wild [18]. Ayele et al*.* [19] reported that colony mosquitoes fed more efficiently, exhibiting infection intensities approximately twice as high as those observed in field-derived mosquitoes.

This study presents an alternative MFA method that eliminates the need for a circulatory water bath, glass membrane feeders, complex tubing systems, and metal clamps. This simplified approach enables MFAs to be conducted in a diverse range of settings, including field studies and laboratories in resource-limited countries.

## Methods

### *P. falciparum* in vitro culture

*P. falciparum* NF54 parasites were cultured according to the method described by Trager and Jenson [20]. Briefly, parasites were initiated at a parasitemia of 0.3% and 4% hematocrit in RPMI media supplemented with 10 µg/ml hypoxanthine, 25 mM HEPES, and 10% normal human serum. Parasites were maintained at 37 °C using the candle jar method to provide microaerophilic conditions for optimal growth. Mature stage gametocytes were harvested after development in culture for 15–17 days. Parasitaemia was determined and adjusted to 0.3% mature gametocytaemia or as otherwise stated before it was used to feed *Anopheles* mosquitoes.

### Mosquito maintenance and infection by membrane feeding assays

**Fig. 1:**
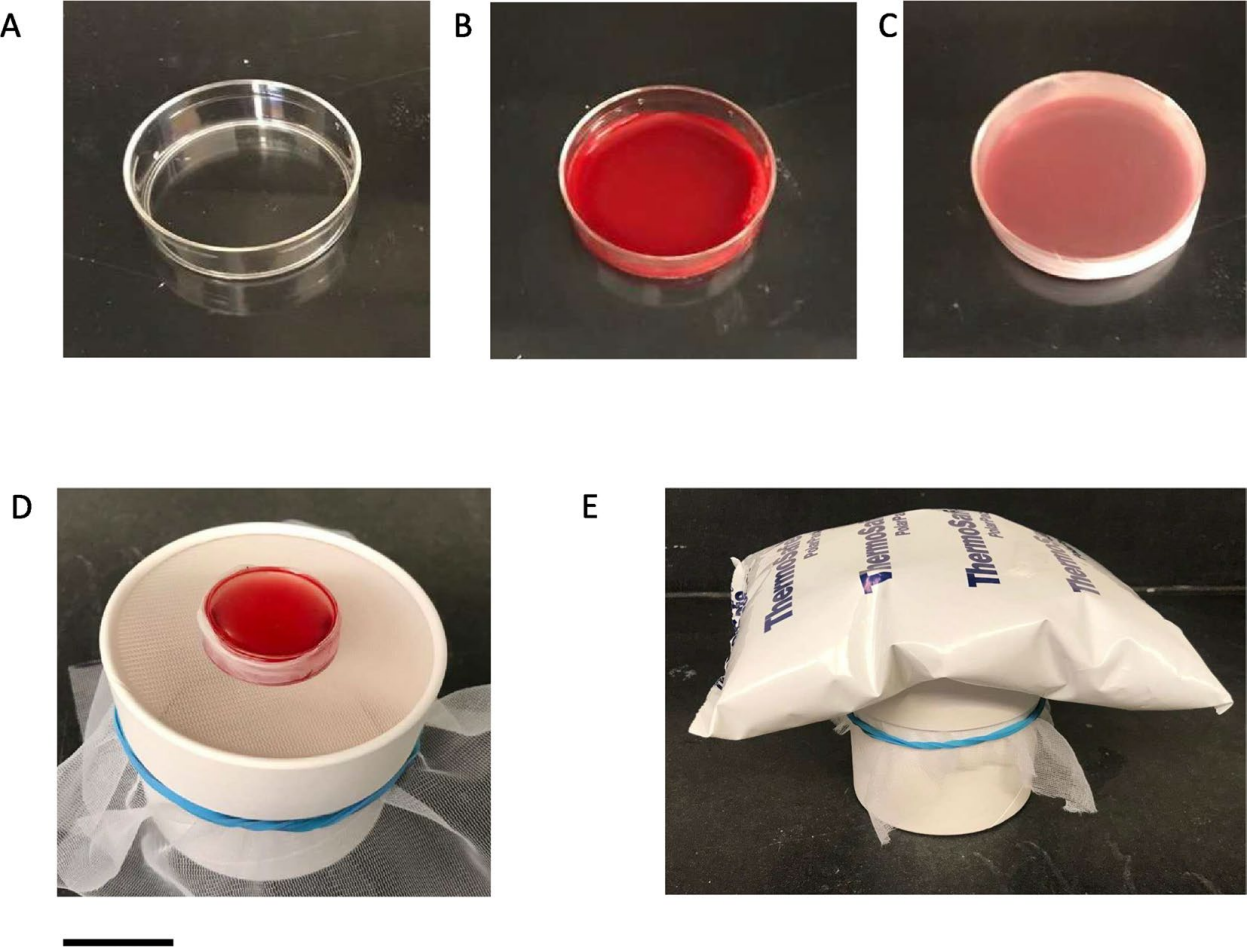
Setting up mosquito membrane feeding assays using a Petri dish kept warm by a gel warmer. **A** A 35 mm Petri dish is placed on a slide warmer. **B** An infectious blood meal (500 µl) is added to the Petri dish. **C** the Petri dish is covered with a parafilm membrane. **D** The Petri dish is gently swirled to spread the gametocyte culture and is turned upside down and placed on top of a mosquito cup. **E** A pre-warmed gel pack is placed on top of the Petri dish and mosquitoes are allowed to feed for 30 min. Scale bar = 1 in.

*Anopheles* mosquitoes were maintained at 27 °C and 80% relative humidity under a 14-h light and 10-h night cycle. Mosquitoes were fed with 10% sucrose solution. Before conducting blood feeds, 4–6 day old *An. stephensi* (Liston strain) or *An. gambiae* (Keele strain) mosquitoes were starved for 6 h by removing sucrose. Female mosquitoes were transferred to one-pint paper cups with each cup containing approximately 60 mosquitos. The blood feedings were conducted under three different conditions described below. The first (1) condition that acted as control was feeding mosquitoes with gametocytes using the standard glass membrane feeders connected to a circulatory water bath set at 37 °C as previously described [12]. For the second (2) feed, 500 µl of gametocytes diluted to 0.3% were added to a 35 mm × 10 mm Petri dish (Corning 430135) and placed on top of a slide warmer. The Petri dish containing blood was covered with stretched-out parafilm. Before placing the Petri dish on top of a mosquito cage, it was gently swirled to spread the blood and inverted so that the side with parafilm was in contact with the netting. A prewarmed (at 37 °C) Thermosafe (Sonoco Thermosafe, USA) gel warmer was placed above the Petri dish to keep the blood meal warm (see Fig. 1)**.** Temperature measurements (°C) were taken at 5 min intervals to monitor the stability of temperature while mosquitoes obtained an infectious blood meal by inserting a long thermometer probe under the gel heat pack. Under the third (3) condition, a Petri dish containing gametocytes prepared as previously described was placed on top of a mosquito cage without a gel warmer, and the mosquito cage was placed in a 37 °C incubator. The same blood volume and gametocyte concentration were used under the three different conditions described above.

Mosquitoes were allowed to feed for 30 min after which they were knocked down by exposure to 4 °C for about 5–10 min. Unfed mosquitoes were removed and counted to determine the % that took a blood meal. Fed mosquitoes were returned into paper cups, provided a 10% sucrose meal, and incubated at 25 °C for 7–8 days. Seven days post blood-feeding, mosquito midguts were dissected and stained with 0.1% mercurochrome for 2 min. Midguts were visualized under the light microscope at 100X magnification. The prevalence of infection was determined by counting midguts that had at least one oocyst. Due to the high infection intensity observed in *An. gambiae*, oocyst counts for these mosquitoes were capped at a maximum of 300 oocysts per midgut to prevent counting errors. The intensity of infection was determined by counting the number of oocysts per individual midgut for all mosquitoes fed under the three experimental conditions.

### Simplified MFA works with major malaria vectors *An. stephensi* and *An. gambiae*

After demonstrating that an infectious blood meal could be successfully introduced to mosquitoes through a parafilm-covered Petri dish where the temperature was maintained by a gel warmer, we went on to assess whether this method could be applied to more than one species of *Anopheles* mosquitoes*.* We conducted membrane-feeding experiments with either *An. stephensi* or *An. gambiae* starved for 6 h under conditions 1 and 2, i.e., SMFA using glass membrane feeders connected to a circulatory water bath or gel warmer-covered Petri dishes. Oocyst dissections were done 7–8 days post blood feeding as previously described.

The statistical significance of differences in oocyst numbers in mosquitoes fed under different membrane-feeding assay conditions was analysed by the Mann–Whitney test, and *P* values < 0.05 were considered statistically significant.

## Results

### Simplified MFA with gel warmers for temperature maintenance, performs comparably to traditional SMFA

**Fig. 2:**
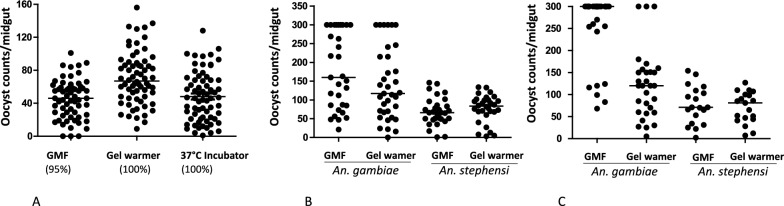
**A** Comparison of oocyst counts from mosquitoes that obtained infectious blood meals under three different conditions, using glass membrane feeder (GMF) (*n* = 61), gel warmer (*n* = 66) and 37 °C Incubator (*n* = 67). *An. stephensi* mosquitoes were starved and fed with infectious gametocytes at 0.3% gametocytemia. Midguts were dissected 7 days post-blood feeding and stained with 0.1% mercurochrome. The total number of mosquitoes dissected is indicated by *n*, and oocyst medians are indicated by horizontal bars. Prevalence (shown in parenthesis) was expressed as the number of mosquitoes with at least one oocyst over the total number of mosquitoes dissected. Data is pooled from two independent experiments. Statistical analyses were performed using the Mann–Whitney test to compare oocyst counts of mosquitoes fed under three different conditions.** B** Infection prevalence and intensity within each species were similar for mosquitoes fed using GMF or Petri dishes/gel warmers at 0.3% gametocytemia. *An. stephensi* or *An. gambiae* mosquitoes were starved and fed with infectious gametocytes at 0.3% (**B**) or 0.15% (**C**) gametocytemia. Each dot on the graph represents an individual mosquito midgut. Median oocyst counts are indicated with a horizontal bar in each column. Results (**B**) are representative of three replicates. Statistical analyses were performed using the Mann–Whitney test to compare oocyst counts within each species. Within species differences in median oocyst counts were not statistically significant (*P* > 0.05) when using 0.3% gametocytemia

*An. stephensi* mosquitoes were fed under three different conditions i.e. standard membrane feeding assay using glass membrane feeders, at room temperature with a gel warmer covering a 35 mm Petri dish containing blood, or with a Petri dish placed in a 37 °C incubator. Oocyst counts from mosquitoes fed under the three different conditions were compared as shown in Fig. 2A. This figure represents pooled data from two independent experiments. The prevalence of infection was 100% in mosquitoes fed via a Petri dish covered with a gel warmer or mosquitoes placed inside a 37 °C incubator (Fig. 2). This prevalence was comparable to control mosquitoes fed using glass membrane feeders (95%). These results demonstrate that membrane feeding assays can be done using a Petri dish and gel warmers or with a Petri placed inside a 37 °C incubator thus obviating the need for glass membrane feeders connected to a circulatory warm water bath.

Mosquitoes fed in a 37 °C incubator produced oocyst counts similar to mosquitoes fed using glass membrane feeders (median 46) see Fig. 2A. Mosquitoes fed with blood warmed by a gel pack produced the highest number of oocysts (median oocyst count, 67) per midgut and this difference was statistically significant when compared to mosquitoes fed either through the glass membrane feeder or to mosquitoes fed inside a 37 °C incubator (Mann–Whitney test *p* < 0.05). Our results also showed that the proportion of starved mosquitoes taking a blood meal was similar under a Petri dish with gel warmers (80%, *n* = 65) compared to feeding by glass membrane feeders (88%, *n* = 72). The proportion of mosquitoes taking a blood meal in a 37 °C incubator was within the range (82%, *n* = 72) of mosquitoes fed under a Petri dish with gel warmers or by glass membrane feeding. To assess the stability of the gel pack's temperature during the 30-min feeding period, measurements were taken and consistently ranged between approximately 34 and 35 °C (see Table 1). Notably, the temperature remained stable for up to 1 h, staying above 33 °C, well above the 30 °C threshold which triggers gametocyte activation [21].

**Table 1:** The temperature stability of prewarmed gel warmers was measured for 1 h during blood feeding at room temperature

Time (min)	Temperature °C
5	35
10	35
15	35
20	34.5
25	34.5
30	34.2
35	34
40	34
45	33.8
50	33.5
55	33
60	33

Temperature (°C) measurements were taken at 5 min intervals, and the results are representative of at least two independent experiments

### Simplified MFA works with major malaria vectors, *An. stephensi* and *An. gambiae*

After demonstrating that mosquitoes fed using a Petri dish either at 37 °C or covered with a gel warmer at room temperature produced oocysts numbers comparable to mosquitoes fed using glass membrane feeders. Next, we assessed whether this simplified and low-cost method (using a 35 mm Petri dish plus gel warmer) could be used for different *Anopheles* mosquito species. Feeds were performed under two conditions, i.e., glass membrane feeders or Petri dishes with gel warmers using either *An. stephensi* or *An. gambiae* mosquitoes*. An. stephensi* and *An. gambiae* mosquitoes produced similar numbers of oocysts when fed either by glass membrane feeders or by using a Petri dish/gel warmer combination (Fig. 2B). However, *An. gambiae* had a higher intensity of infection compared to *An. stephensi* (Fig. 2B,) under the two methods used. The median oocyst counts for *An. gambiae* mosquitoes fed using a Petri dish plus gel warmer was 117 whereas for *An. stephensi* the median was 83. The median number of oocysts in *An. gambiae* (160 oocysts) were more than two-fold higher than oocysts produced in *An. stephensi* (66 oocysts) when comparing mosquitoes fed using glass membrane feeders*.* This difference in infection intensity between *An. gambiae* and *An. stephensi* was consistent even when a lower concentration of gametocytes (0.15%) was used (panel C). In this experiment (Fig. 2C) a difference was observed in the infection intensity between *An. gambiae* mosquitoes fed using the glass membrane feeder compared to mosquitoes fed under the Petri dish/heat gel pack method. *An. gambiae* mosquitoes fed via glass membrane feeder had a mean oocyst count of 257 compared to 121 for mosquitoes fed using the Petri dish/heat gel method. This variation in infection intensity was observed exclusively with *An. gambiae* and not with *An. stephensi*.

## Discussion

Malaria control will require a concerted effort and transmission-blocking interventions being developed can play an important role in reducing malaria transmission. Membrane feeding assays are critical to study naturally acquired immunity to sexual stages of *Plasmodium* [22, 23] as well as the development of transmission-blocking drugs [24], vaccines [25] and evaluation of transgenic mosquitoes refractory to *Plasmodium*. SMFAs have traditionally relied on jacketed glass membrane feeders that are coupled to a circulatory water bath. This configuration necessitates hand-made glass membrane feeders, a circulatory warm water bath to keep the blood meal at 37 °C, intricate tubing connections, and clamps to secure the glass feeders on top of mosquito cages. When conducting multiple assays simultaneously or performing assays in a field setting or for laboratories in low-resource countries, SMFA using glass membrane feeders can be very challenging.

The current study reports a membrane-feeding assay that uses a 35 mm Petri dish covered with parafilm where the temperature of the blood meal is maintained by a gel warmer placed on top of the Petri dish (Fig. 1). These findings demonstrate that the new method described here yields comparable levels of oocyst infection intensity compared to traditional glass membrane feeders (Fig. 2A). This suggests that using 35 mm Petri dishes and warm gels can be a simpler and less expensive alternative for conducting SMFAs. While developing the simplified MFA, it was also observed that mosquitoes can be blood-fed in a 37 °C incubator and develop infection intensity comparable to SMFAs. We opted to utilize gel warmers for feeding at room temperature to avoid the potential impact on mosquito physiology at warmer temperatures. It is important to consider whether allowing mosquitoes to feed in a controlled 37 °C incubator accurately reflects their natural feeding conditions. Furthermore, research has indicated that mosquitoes remain unaffected by ambient temperatures up to 33 °C. However, once temperatures surpass this threshold, mosquito behavior is impacted negatively during blood feeding [26].

Apart from the cost of using glass membrane feeders for SMFA, performing multiple feeds simultaneously becomes cumbersome as it requires intricate tube connections to a circulatory water bath. Recent efforts are exploring ways to mitigate the cost of membrane feeders. It has been demonstrated that membrane feeders can be produced through 3D printing technology that costs a fraction of glass membrane feeders [27, 28]. Even though the cost can be reduced by up to a third, it remains necessary to connect these 3D-printed membrane feeders to a water bath using tubes and clamps. The method presented here which uses Petri dishes and reusable gel warmers greatly simplifies performing membrane feeds, whether performing multiple feeds in a laboratory or performing DMFA in a field setting. The Petri dish approach also presents a distinct advantage over the Hemotek method where only up to 6 mosquito cages can be fed simultaneously [29].

Several other simple and low-cost alternatives to mosquito membrane feeding have been described in the literature. For example, conical tubes filled with prewarmed (50 °C) glycerol (Glytube) and covered with parafilm have been used to blood feed *Aedes aegypti* mosquitoes [30]. Mosquito blood feeds have been performed by utilizing disposable Styrofoam cups filled with warm water and covered at the bottom with a polytetrafluoroethylene-based membrane [31]. However, both methods described above have not been evaluated in studies using infectious blood meals to *Anopheles* mosquitoes. It remains to be determined how glycerol prewarmed to 50 °C affects *Plasmodium* parasites. Additionally, the Glytube method can present a challenge under field settings where glycerol, Dura Seal film and an apparatus to prewarm glycerol to 50 °C may not be readily available.

In this study, disposable plastic Petri dishes were used for blood feeding. However, it is worth noting that glass Petri dishes are also a viable option. Glass Petri dishes can be easily sterilized after each use, allowing for re-use and increasing cost-effectiveness. The gel warmers can also be reused several times for multiple blood feeding experiments.

The simplified MFA described in this study will be particularly useful in the evaluation of transmission potential of patients infected with malaria parasites. In most cases, mosquito rearing facilities where SMFA or DMFA can be performed, are far from the field sites/health facilities, where blood samples are drawn and collected. To prevent premature gametogenesis, venous blood must be maintained at human body temperature. A recent method described using a thermos flask to keep gametocyte infected blood samples warm for up to 4 h before samples can reach the laboratory where DMFA can be performed [17].

The approach proposed here can facilitate DMFAs in the field, allowing samples collected from patients to be quickly assessed for mosquito transmissibility without the need of transportation to a laboratory. It should be noted that a 37 °C incubator was used to prewarm the gel heat packs, and this may present a challenge in the field where electricity to run an incubator may not be available. This limitation can be addressed by using available heat sources, such as heated water, to warm the gel packs. Thermos flasks can then be employed to maintain the gel packs at the required temperature until blood samples are ready for mosquito feeding. Alternatively, commercially available heat packs that do not require pre-warming, such as Uniheat packs, can be used for feeding experiments. These packs can maintain warmth for up to 72 h, providing a practical solution in field settings [32].

## Conclusion

A simplified approach has been established to perform membrane feeding assays utilizing 35 mm Petri dishes. To ensure the optimal temperature for blood feeding is maintained, gel warmers were placed atop Petri dishes during mosquito blood feeding. Major malaria vectors, *An stephensi,* and *An. gambiae* infected using the simplified MFA showed oocyst infection intensity and prevalence comparable to mosquitoes infected using SMFA. The method described here is simple, easy to set up, requires inexpensive consumables, can be easily adapted to laboratories with limited resources, and applied under field settings.

## Data Availability

Data is provided within the manuscript or supplementary information files.

## Abbreviations

SMFA: Standard membrane feeding assays
MFA: Membrane feeding assays
DMFA: Direct membrane feeding assays
